# Conventional versus high-voltage, long-term pulse Radiofrequency of ganglion impar in perineal pain with advanced rectal cancer: a Randomized, double-blind controlled trial

**DOI:** 10.1186/s12871-024-02717-0

**Published:** 2024-09-12

**Authors:** Qin Li, Huaiming Wang, Bo Zhong, Taomei Zhang, Zhiqiang Wang, Ping Tao, Jiang Zou, Aimin Zhang

**Affiliations:** 1 Department of Anesthesiology, Sichuan Clinical Research Center for Cancer, Sichuan Cancer Hospital & Institute, Sichuan Cancer Center, Affiliated Cancer Hospital of University of Electronic Science and Technology of China, Chengdu, China; 2 Department of Anesthesiology, Yanjiang District People’s Hospital, Ziyang, 641399 Sichuan China

**Keywords:** Ganglion impar, Conventional radiofrequency, Pulsed radiofrequency, Perineal pain, Cancer pain

## Abstract

**Study objective:**

Advanced rectal cancer is a common cause of perineal pain and research on the use of radiofrequency therapy for the treatment of this pain is limited. In the present study, we aimed to compare the effectiveness and safety of conventional radiofrequency (CRF) and high-voltage long-term pulsed radiofrequency (H-PRF) of radiofrequency therapy in the management of perineal pain in advanced rectal cancer.

**Design:**

Randomized, Double-Blind Controlled Trial.

**Setting:**

Sichuan Cancer Hospital & Institute and Yanjiang District People’s Hospital in Sichuan, China.

**Participants:**

A total of 72 patients with advanced rectal cancer experiencing perineal pain who were accepted for radiofrequency treatment.

**Interventions:**

Patients were assigned randomly (1:1) assigned to either the group CRF or H-PRF in a double-blind trial.

**Measurements and main results:**

The primary focus was on assessing perineal pain using numeric rating scales (NRS) scores at various time points. Secondary outcomes included the duration of maintaining a sitting position, depression scores, sleep quality, consumption of Oral Morphine Equivalent and Pregabalin, and the incidence of perineal numbness. A total of 57 patients (28 patients in the group CRF and 29 patients in the group H-PRF) were investigated. At all observation time points postoperatively, both groups of patients exhibited significant reductions in pain, enhancements in depression, improvements in sleep quality, and increased duration of sitting compared to their baseline measurements (*P*<0.05). During the 3 months and 6 months follow-up period, the group CRF exhibited significant reduction in pain, improvement in depression, sleep quality, and increased the time of keeping a sitting position compared with the group H-PRF (*P*<0.05). The consumption of oral morphine equivalent and Pregabalin as well as the incidence of perineal numbness were not significantly different between groups (*P* > 0.05).

**Conclusion:**

Our results demonstrate that application of CRF and H-PRF in ganglion impar to reduce perineal pain and improve the quality of life of patients with advanced rectal cancer is safe and effective. However, the long-term effect of CRF is better compared with that of H-PRF.

**Trial registration:**

https://www.chictr.org.cn/ (ChiCTR2200061800) on 02/07/2022. This study adheres to CONSORT guidelines.

**Supplementary Information:**

The online version contains supplementary material available at 10.1186/s12871-024-02717-0.

## Introduction

Pain is a prevalent symptom among patients with advanced diseases, with approximately 59% of patients undergoing cancer treatment, 64% of patients with advanced disease, and 33% of patients receiving curative treatment reporting pain [1]. In addition, 10–20% of cancer patients may experience refractory cancer pain [2]. Pain associated with advanced rectal cancer typically manifests in the perineum, abdomen, and lower back, with perineal pain being particularly pronounced. This discomfort significantly impacts the quality of life of patients [3].

The ganglion impar is located anterior to the articulation of the sacrum and coccyx, and it is innervated by sympathetic and parasympathetic nerve fibers originating from the lumbosacral region. It modulates the transmission of signals related to nociceptive pain and sympathetic nerve pain in the perineum [4].The ganglion impar block (GIB), also known as Walters’ block, is an easy and efficacious technique for targeting pelvic and perineal nociceptive pathways [5], GIB has demonstrated good efficacy in managing perineal pain [6]. The clinical application of GIB has been extended to include drug blocking, chemical damage, and radiofrequency therapy [7]. Accumulating evidence indicates that Ganglion Impar Radiofrequency (RF) can alleviate cancer-related and non-cancer-related pain [7, 8].

There are two modes of radiofrequency: conventional radiofrequency (CRF) and pulsed radiofrequency (PRF). CRF is safe and effective in treating sympathetic ganglia, whereas PRF exhibits limited therapeutic efficacy [8]. Following the application of the ganglion impar high-voltage long-term pulsed radiofrequency (H-PRF) regimen, the remission rate of pudendal neuralgia remained high at 88.6% even after 3 months [9], making it a promising strategy for the treatment of cancerous perineal pain [7]. Nevertheless, few studies have directly compared the effectiveness of CRF versus H-PRF in the management of cancer-related perineal pain.

The aim of this study was to evaluate the efficacy and safety of CRF and H-PRF of the ganglion impar in the management of perineal pain associated with advanced rectal cancer.

## Methods

### Study design

This pilot study, conducted at the Department of Anesthesiology at Sichuan Cancer Hospital & Institute and Yanjiang District People’s Hospital, was a randomized, double-blind controlled trial registered with the Chinese Clinical Trial Registry (ChiCTR2200061800). The study was approved by the Medical Ethics Committee of the Ziyang People’s Hospital and Sichuan Cancer Hospital & Institute (KY-sj-2023-02) and all procedures were carried out in compliance with the Declaration of Helsinki. Prior to any procedures, written informed consent was obtained from the participants. There were no changes to methods or trial outcomes after the trial commenced and no unintended effects as a result of the trial.

### Randomization

To achieve a double-blind procedure, two physicians attended the participants at each center. One physician conducted assessments while the other managed randomization and device instructions. Subjects were informed about potential sensations during treatment and instructed not to disclose them to the assessing physician. A clinical nurse who was not involved in the process of designing the protocol, assigned randomization numbers and allocated 72 patients into either the group CRF or H-PRF at a 1:1 ratio. The treatment allocation was conducted in line with a predetermined randomization list generated using random blocks.

### Subjects

Both male and female patients attending the pain clinic were assessed to determine their eligibility and those that met the criteria were asked to participate in the study. The inclusion and exclusion criteria were assessed within the Department of Anesthesiology.

### Inclusion criteria

Pathologically confirmed diagnosis of rectal cancer,

The cancer-associated pain experienced by the subject in other regions remains within an acceptable threshold, as indicated by a NRS score of 3 or below,

Cancerous pain with perineal pain,

Ineffective perineal pain relief after oral opioid analgesics combined with pregabalin, willingness to accept invasive interventions,

Positive symptoms of the Grading System for Neuropathic Pain (GSNP) with a score of 3 or 4 [10],

Life expectancy of at least 6 months,

Perineal pain with the following characteristics: (1) the pain was significantly Aggravated when sitting down, (2) the pain did not affect sleep at night, (3) the pain was not accompanied by objective sensory impairment, (4) the pain in the anal area was reduced after the diagnostic nerve block of the azygous ganglion.

### Exclusion criteria

Lumbosacral fracture or local anatomical variation that makes it difficult to puncture,

Systemic bacteremia or local infection at the puncture site,

There are symptoms such as coagulation system diseases, diabetes, peptic ulcers, infections, and mental and psychological diseases,

Developed recent myocardial infarction, severe bradyarrhythmia, or heart block.

### Intervention

**Fig. 1:**
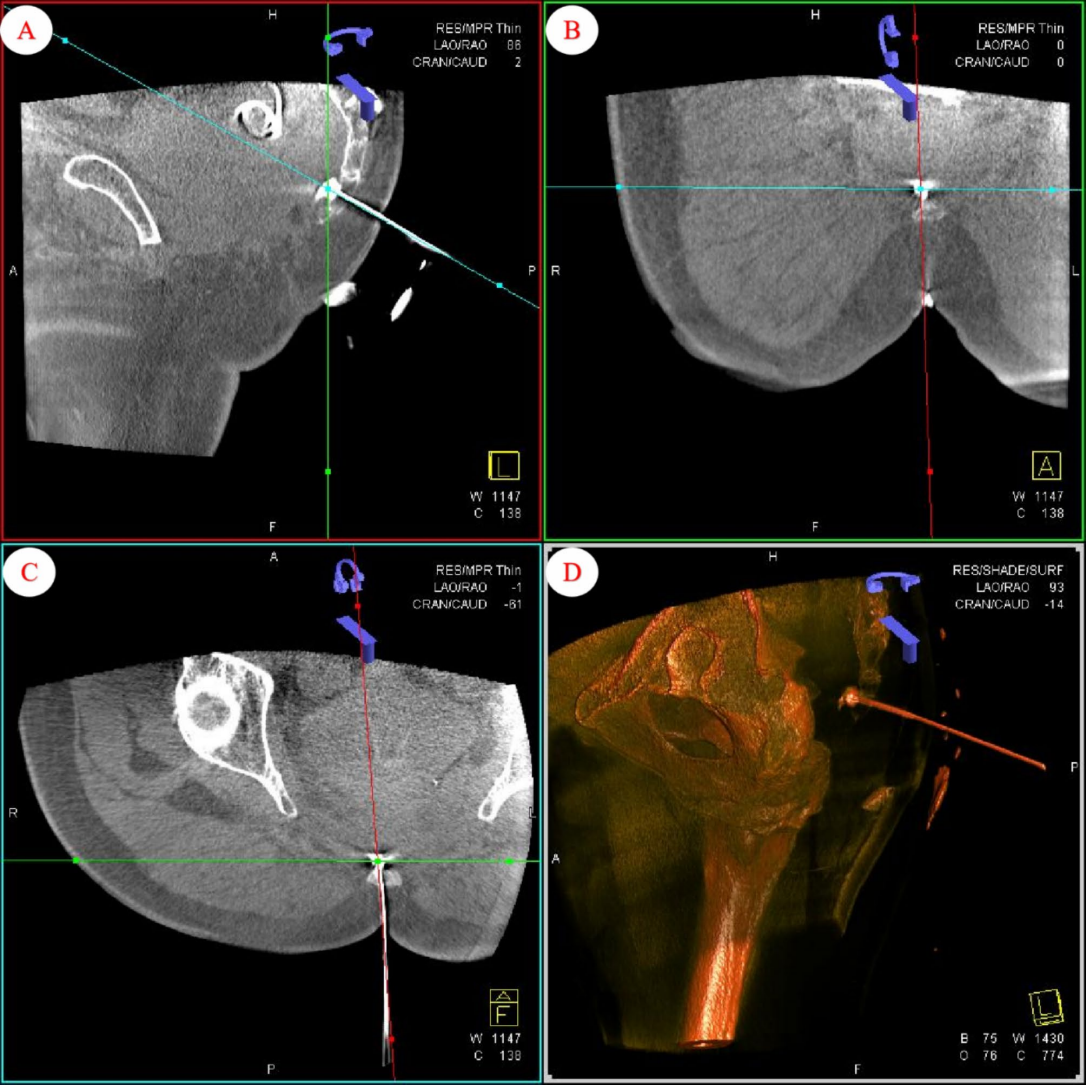
The tip position of the needle was determined after the XperCT image reconstruction of DSA. **A**: In the sagittal plane, the needle tip of the puncture trocar is observed to be situated in the space between the anterior aspect of the coccyx and the rectum, with no evidence of contrast agent infiltration into the rectum or blood vessels. **B**: The coronal view reveals the puncture trocar needle traversing the first coccygeal joint space. **C**: In the cross-sectional view, the contrast agent is seen to diffuse between the anterior aspect of the coccyx and the rectum. **D**: Three-dimensional imaging elucidates the spatial relationship between the puncture trocar and the surrounding tissues(-)

Once patients were admitted to undergo intervention surgery, standard indoor monitoring procedures were implemented. These procedures involved assessing the patient’s blood pressure, heart rate, respiration, pulse, and pulse oxygen saturation. The patient was then positioned in a prone position on the surgical treatment bed, and the disinfected following standard protocols. Next, the sacral and coccyx regions were visualized using oblique fluoroscopy to identify the needle insertion point, specifically the first coccyx joint space. 2 ml of 1.0% lidocaine was administered for local anesthesia before needle insertion under oblique fluoroscopy. With the assistance of DSA, the needle tip of the puncture trocar is precisely inserted through the first coccygeal joint. Finally, following the injection of 0.5 ml of contrast agent and the reconstruction of the XperCT image, the needle tip was repositioned behind the rectum and in front of the anterior longitudinal ligament of the coccyx. (refer to Fig. 1).

Subsequently, the central needle of the trocar is withdrawn, and the radiofrequency electrode is positioned within the trocar. The therapeutic effects are achieved through the thermal and electric fields generated by the electrode tip. Radiofrequency Electrode Position Test: Upon determining the needle tip location, a stimulation protocol of 50 Hz at 0.1–0.6 V was administered to elicit discharge-like pain within the ganglionic innervation area. This was followed by a secondary stimulation at 2 Hz and 0.5-2.0 V, aimed at achieving consistent non-contraction of the perineal muscles. Successful targeting of the electrode position was confirmed through these responses.

Patients in the group CRF underwent a continuous opening procedure starting at 50 °C for 30 s, followed by 55 °C for 30 s, with a gradual increase in temperature to 60–70 °C for 60 s, 75 °C for 120 s, and 80 °C for 180 s. After surgery, approximately 4 ml of 0.5% ropivacaine was administered to alleviate pain associated with local heat injury. In the H-PRF group, the radiofrequency instrument was re-configured to the manual pulse mode with specific parameters including a temperature of 42 °C, frequency of 2 Hz, pulse duration of 20 ms, time duration of 900 s, and an initial pulsed RF field strength of 40 V which was incrementally raised to the maximum tolerable level by the patient (up to 100 V). The patient was monitored for 10 min post-treatment for abnormalities. Subsequently, they were returned to the ward where vital signs were also monitored using an electrocardiogram. The patient was instructed to lie on their back for 3 h, and changes in lower limb blood flow were observed.

### Collected data

The data collection and patient assessment were conducted by a well-trained pain physician who was blinded to the technique utilized. During subsequent visits, patients were prohibited from reviewing their previous data.

### Assessments

The patients were evaluated at multiple time intervals, including baseline (pre-surgery), 24 h following surgery, 1 week after surgery, 1month post-surgery, 3 months post-surgery, and 6 months post-surgery.

The demographic data collected included age, weight, Body Mass Index (BMI), duration of the pain procedure, initial numeric rating scales (NRS), GSNP, initial total daily dose of morphine and pregabalin, and duration of medication treatment.

#### A) primary outcome

Pain intensity was evaluated using the Numerical Rating Scale (NRS), which ranged from 0 to 10. A 10 cm scale was employed to measure pain levels, with zero representing the absence of pain and 10 representing the most severe pain [11].

#### B) secondary outcome

1) The Patient Health Questionnaire-9 (PHQ-9) self-rating scale was employed to evaluate the severity of depression, consisting of nine items [12]. Despite its brevity, the scale demonstrated good reliability and validity. Scores ranging from 5 to 10 indicated mild depression, 10 to 15 indicated moderate depression, 15 to 20 indicated moderately severe depression, and scores exceeding 20 indicated severe depression [13].

2) The Pittsburgh Sleep Quality Index (PSQI) has been widely applied in the assessment of sleep quality among patients with sleep disorders and is commonly utilized to evaluate sleep patterns in individuals experiencing pain [14]. Questionnaire data revealed that scores on the PSQI ranged from 0 to 5, indicating good sleep, 6 to 10 representing fair sleep, 11 to 15 indicating average sleep, and 16 to 21 corresponding to poor sleep [15].

3) The duration of maintaining a sitting position was recorded as the period within which a patient remained seated in a comfortable position until the onset of anal pain and the subsequent inability to maintain a seated position. These data were monitored by nurses during the patient’s hospital stay period, and family members were trained to perform accurate recording during the post-hospitalization follow-up period [16].

4) The opioid dosage administered preoperatively was quantified by converting it to morphine equivalent units, and the dosage of pregabalin was determined. The occurrence of anal numbness post-treatment, as well as any additional complications, was documented.

### Sample size calculation

Due to limited epidemiological data on the prevalence of rectal cancer presenting with perineal pain, this study was exploratory in nature and did not involve sample size calculations.

### Statistics

Satistical analysis was performed using the SPSS 22.0 software (IBM, Beijing, China, 2017). Normally distributed continuous data were presented as mean ± standard deviation (Mean ± SD). Independent samples t-test was employed to compare two groups, while repeated measures ANOVA was conducted to compare multiple groups. Non-normally distributed continuous data were summarized using the median (M) and interquartile range (IQR). Group comparisons were conducted using the rank sum test, while count data were analyzed using the chi-square test or Fisher exact probability method. Rank data were compared using the rank sum test. A *P*-value < 0.05 was considered to be statistically significant.

## Results

### Patent and clinician characteristics

**Fig. 2:**
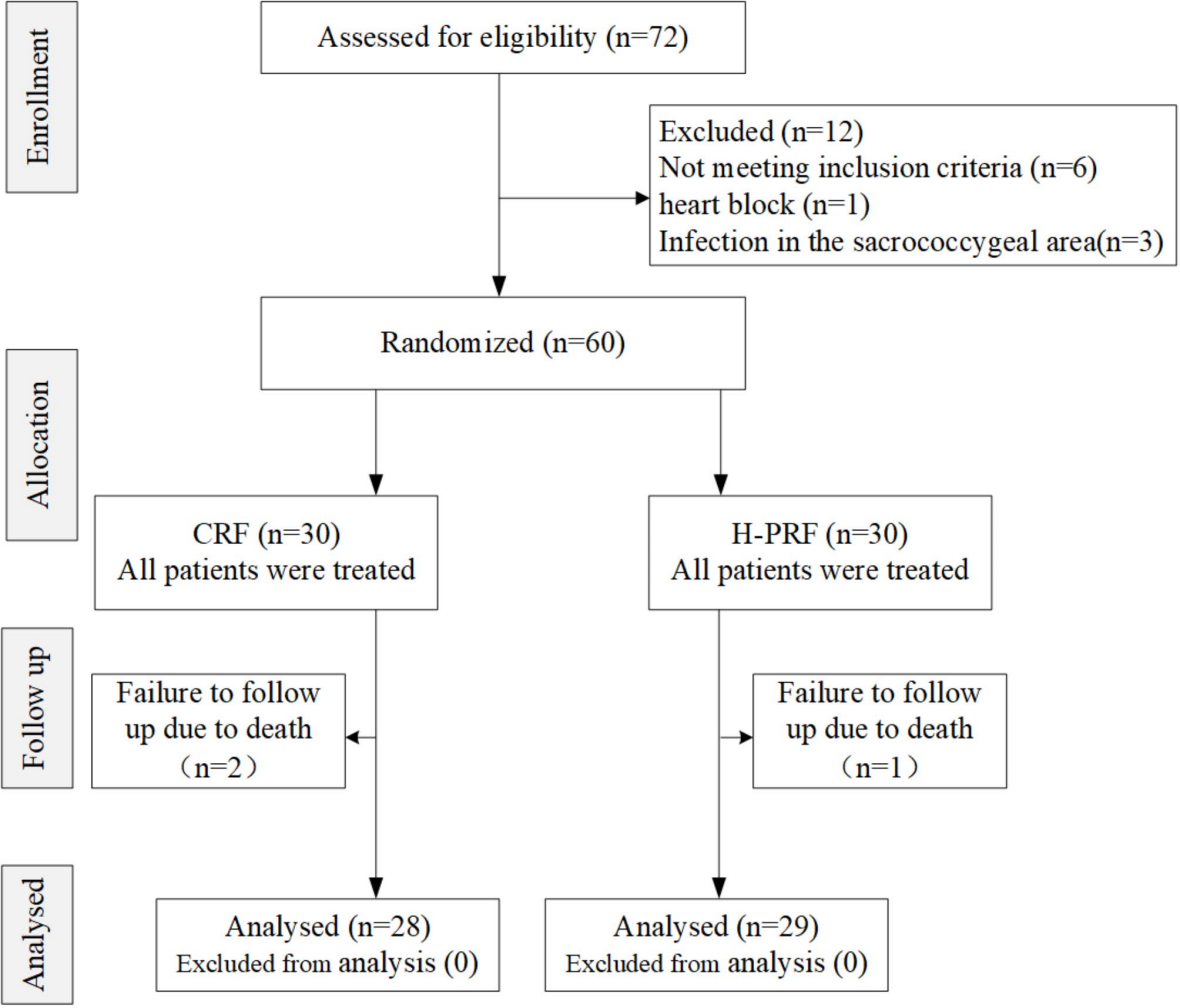
CONSORT flow diagram showing selection of study participants

Initially, 72 patients were screened at the Sichuan Cancer Hospital & Institute and Yanjiang District People’s Hospital. Among them, 12 individuals were excluded. Six cases were excluded due to failure to meet inclusion criteria, two cases due to the presence of heart block, and three cases due to sacrococcygeal area infection. Consequently, 60 patients were enrolled in the study, with three individuals withdrawn due to lack of follow-up resulting from mortality. Consequently, the final analysis included 57 patients, consisting of 32 males and 25 females within the cohort (Fig. 2), the patient characteristics are outlined in Table 1.

**Table 1:** Characteristics of the patients

		CRF (*n* = 28)	H-PRF (*n* = 29)	t/χ^2^-value	*P*-value
Age (years), mean ± SD		68.9 ± 6.9	70.3 ± 7.1	-0.764	0.448
Sex, n (%)				0.147	0.186
Male		13 (46.4)	19 (65.5)		
Female		15 (53.6)	10 (34.5)		
Weight (kg), mean ± SD		54.5 ± 5.5	53.8 ± 6.8	0.426	0.671
BMI (kg/m^2^)				0.453	0.797
<18.5		7 (25.0)	8 (27.6)		
18.5 ~ 24		12 (42.9)	14 (48.3)		
>24		9 (32.1)	7 (24.1)		
Pain duration (month)				1.233	0.540
<3		3 (10.7)	6 (20.7)		
3 ~ 6		9 (32.1)	7 (24.1)		
>6		16 (57.2)	16 (55.2)		

### Efficacy

The NRS scores significantly decreased at the intervals of 24 h, 1 week, 1 month, 3 months, and 6 months post-procedure compared to baseline in both groups (*P*<0.001). The NRS scores were significantly lower at the 3-month (*P*<0.002) and 6-month (*P*<0.005) time points in group CRF compared to group H-PRF. Notably, there were no significant differences in pain levels between the two groups before the procedure, and at the 24-hour, 1-week, and 1-month follow-up assessments. Table 2 shows that patients in group CRF had better analgesic outcomes compared to those in group H-PRF, as indicated by repeated measures analysis of variance (*P* < 0.001).

**Table 2:** NRS, PHQ-9, PSQI and the time of keeping a sitting position of the studied groups

	CRF (*n* = 28)		H-PRF(*n* = 29)	t/χ^2^-value	*P*-value
NRS					
pre	6.54 ± 1.95		6.81 ± 1.42	-0.599	0.552
After 24 h	4.33 ± 0.37^**^		4.46 ± 0.52^**^	-1.084	0.283
After 1 week	2.41 ± 0.46^**^		2.45 ± 0.77^**^	-0.237	0.814
After 1 month	1.55 ± 0.41^**^		1.47 ± 0.61^**^	0.579	0.565
After 3 months	2.56 ± 0.67^**^		3.23 ± 0.87^**^	-3.249	0.002
After 6 months	2.98 ± 0.72^**^		3.46 ± 0.52^**^	-2.893	0.005
Time effect				*F* = 455.156, *P*<0.001	
Time and group interaction effects				*F* = 37.983, *P*<0.001	
PHQ-9					
pre	10.32 ± 1.18		10.98 ± 2.17	1.409	0.164
After 24 h	6.24 ± 1.86^**^		6.43 ± 1.39^**^	-0.438	0.663
After 1 week	7.22 ± 2.01^**^		7.17 ± 1.16^**^	0.116	0.908
After 1 month	7.48 ± 1.95^**^		7.52 ± 0.98^**^	-0.098	0.992
After 3 months	8.03 ± 1.43^**^		9.22 ± 1.14^**^	-3.480	0.001
After 6 months	8.09 ± 1.88^**^		8.87 ± 1.21^**^	-2.588	0.012
Time effect				*F* = 152.5076, *P*<0.001	
Time and group interaction effects				*F* = 19.798, *P*<0.001	
PSQI					
pre	13.6 ± 3.8		14.0 ± 2.7	-0.459	0.648
After 24 h	7.5 ± 1.2^**^		7.8 ± 1.7^**^	-0.767	0.446
After 1 week	5.8 ± 1.5^**^		6.2 ± 0.9^**^	-1.226	0.225
After 1 month	4.7 ± 0.8^**^		5.1 ± 1.2^**^	-1.475	0.146
After 3 months	5.6 ± 1.2^**^		8.5 ± 2.4^**^	-5.738	<0.001
After 6 months	7.1 ± 1.9^**^		8.8 ± 1.8^**^	-4.469	0.001
Time effect				*F* = 193.478, *P*<0.001	
Time and group interaction effects				*F* = 21.354, *P*<0.001	
The time of keeping a sitting position **(min)**					
pre		22.6 ± 7.7	24.1 ± 6.3	-0.798	0.428
After 24 h		58.3 ± 11.9^**^	55.6 ± 8.1^**^	0.992	0.325
After 1 week		63.2 ± 13.6^**^	63.5 ± 11.3^**^	-0.090	0.929
After 1 month		61.1 ± 10.4^**^	62.8 ± 17.4^**^	-0.444	0.659
After 3 months		55.5 ± 11.1^**^	48.8 ± 7.5^**^	2.725	0.009
After 6 months		48.1 ± 8.8^**^	40.9 ± 7.2^**^	-3.351	0.001
Time effect				*F* = 348.157, *P*<0.001	
Time and group interaction effects				*F* = 41.159, *P*<0.001	

Data are presented as mean ± SD, NRS = numerical rating scale. ^*^P<0.05, ^**^P<0.01, ^***^P<0.001, compared to the baseline in each group

Our results indicated that PHQ-9 scores were significantly decreased at the time intervals of 24 h, 1 week, 1 month, 3 months and 6 months post-procedure compared to baseline in both groups (*P*<0.001). Moreover, significantly lowerPHQ-9 scores were recorded at the 3-month (*P*<0.001) and 6-month (*P*<0.012) time points in group CRF compared to group H-PRF. There were no significant differences in depression levels between the two cohorts before the intervention, as well as during the 24-hour, 1-week, and 1-month post-procedure, as depicted in Table 2.

Patients in both groups exhibited enhanced sleep quality as lower pain levels, indicated by a decrease in PSQI scores starting 24 h post-surgery and reaching their lowest point 1-month post-surgery before gradually increasing. Moreover, both groups demonstrated a significant reduction in PSQI scores at all postoperative time points compared to preoperative scores (*P* < 0.05). Further analysis found no statistically significant differences in PSQI scores between the two groups at 24 h, 1 week, and 1month post-procedure (*P* > 0.05). However, the PSQI score of the group H-PRF was significantly higher compared with that of group CRF at 3 months and 6 months post-procedure following the operation (*P* < 0.05), as showed in Table 2.

After receiving treatment, the duration of time spent in a seated position by two groups of patients initially increased within 24 h post-surgery, reached its highest point at 1-week post-surgery, and then gradually decreased. The study found significantly lower sitting-position measurements at both the 3-month (*P* < 0.009) and 6-month (*P* < 0.001) time points in group CRF compared to group H-PRF. Prior to the procedure, as well as at the 24-hour, 1-week, and 1-month follow-up assessments, there were no significant differences in the duration of time spent in a seated position between the two groups (*P*>0.05), as showed in Table 2.

### Oral morphine equivalent and Pregabalin consumption

Patients in both groups experienced rectal and perineal pain, a common consequence of rectal cancer. While radiofrequency therapy successfully reduced perineal pain, oral medications, especially morphine, were still necessary to manage pain in other areas caused by the cancer. Analysis of data shown in Table 3 showed no significant differences (*P* > 0.05) in daily oral morphine equivalent and pregabalin dosage between the two patient groups before and 6 months post-surgery.

**Table 3:** Oral morphine equivalent and Pregabalin consumption of the studied groups

	CRF (*n* = 28)	H-PRF(*n* = 29)	t-value	*P*-value
Oral morphine equivalent (mg)				
pre	375.56 ± 45.82	386.08 ± 50.54	-0.822	0.414
After 6 months	486.81 ± 55.42^***^	505.87 ± 51.36^***^	-1.347	0.183
Pregabalin consumption (mg)				
pre	340.32 ± 51.18	351.85 ± 62.17	-0.081	0.937
After 6 months	358.09 ± 67.82^#^	368.87 ± 71.21^#^	-0.585	0.561

Data are presented as mean ± SD. ^*^P<0.05, ^**^P<0.01, ^***^P<0.001, ^#^*P*>0.05, compared to the baseline in each group

### Safety

Table 4 indicate that neither group of patients experienced adverse events such as significant bleeding at the puncture site, local infection, or rectal injury. Primary postoperative side effect observed was numbness in the perineum. In the CRF group, 16 cases (57.1%) reported perineal numbness 24 h postoperatively, with 6 cases (21.4%) demonstrating persistent symptoms at the 6-month follow-up. Similarly, in the H-PRF group, numbness was reported by 15 cases (51.6%) at the 24-hour mark, with 8 cases (27.5%) still experiencing unresolved symptoms at the 6-month assessment.

**Table 4:** The frequency of adverse events observed within the groups under investigation

	CRF (*n* = 28)	H-PRF(*n* = 29)	χ^2^--value	*P*-value
bleeding	0	0	0	0
local infection,	0	0	0	0
rectal injury	0	0	0	0
perineal numbness				
After 24 h	16 (57.1)	15 (51.7)	0.169	0.792
After 6 months	6 (21.4) ^**^	8 (27.5)	0.292	0.760

## Discussion

In this study, we adopted a double-center, randomized, active-controlled, double-blind clinical trial design to investigate the safety and efficacy of CRF and H-PRF in the treatment of perineal pain and improvement of quality of life in patients with advanced rectal cancer by targeting the ganglion impar. The results demonstrated that the long-term effectiveness of CRF exceeded that of H-PRF.

Occurrence of primary perineal pain has been linked to sympathetic sensory coupling, and the ganglion impar is the distal ganglion of the sympathetic chain [17]. B**l**ocking the ganglion impar can significantly alleviate perineal pain and decrease the need for opioid analgesics [6]. Radiofrequency therapy is an effective technique for the management of ganglion impar. Radiofrequency pain treatment can be categorized into two modes: continuous radiofrequency ablation (CRF) and pulsed radiofrequency (PRF) [18]. Previous studies showed that patients experiencing primary perineal pain can experience a significant reduction in pain over a 6-month monitoring period following treatment with pulsed radiofrequency in the ganglion impar [19]. Nicholas A Zacharias et al. found that PRF and CRF can alleviate sympathetic nerve-mediated pain, including the ganglion impar. Notably, CRF showed better long-term effects in controlling noncancerous perineal pain [20]. In addition, PRF combined with plexus chemical destruction can achieve long-term pain control [21]. The aforementioned studies demonstrate that PRF is still not ideal for long-term relief of perineal pain.

High-voltage pulsed radiofrequency (H-PRF) has a pronounced neuromodulatory effect by generating a robust electric field [7]. Furthermore, H-PRF demonstrates a lasting thermal damage effect, although significantly less pronounced than that observed with CRF techniques. Consequently, H-PRF is an innovative PRF modality that exhibits partial continuous thermal effects and a high-voltage, high-field-strength electric field [22, 23]. Cheng-Long Wang et al. found that H-PRF provided pain relief lasting beyond 12 weeks in individuals diagnosed with pudendal neuralgia, concurrently enhancing mood and overall quality of life [9]. The above results offer compelling evidence confirming that H-PRF treatment yielded significant analgesic effects, alleviated depression, enhanced sitting time, and improved sleep quality in group H-PRF at the six-month follow-up period. In addition, the analgesic efficacy of the H-PRF group was significantly poor compared with that of the CRF group at the 3-month follow-up. This difference may stem from the more precise ablation of the dorsal root ganglion sensory nerve by CRF [23], along with the observation that the duration of pain relief for neuropathic pain with H-PRF using the PRF mode persisted for 3 months [24].

The most common adverse events associated with peripheral nerve radiofrequency therapy are bleeding, local infection, tissue damage, and transient neuritis or neurological deficits, which are influenced by the thoroughness of preoperative assessment and expertise in puncture techniques [25]. Considering that both CRF and H-PRF can increase local temperature to approximately 80 °C, they cause significant damage to pain receptors, thereby reducing pain and yielding a favorable therapeutic outcome. However, it should be noted that this elevated temperature may also induce numbness of the perineal skin in certain postoperative patients [26]. Compared with standard voltage PRF, the incidence of nerve numbness one year post-surgery was higher in the H-PRF group (25% vs. 15%) [27]. Furthermore, the incidence of perineal nerve numbness in the group H-PRF at six months post-surgery was 27.5%, indicating that application of pulsed radiofrequency in high-voltage under long-term mode may result in tissue damage in peripheral nerve as a result of elevated electrode tip temperatures [28].

In our study, a 6-month follow-up revealed that the oral morphine equivalent and pregabalin dosage in the two patient groups did not show a significant decrease, which contrasts with the findings of SOUSA Correia J et al. [6]. Instead, there was a daily increase in morphine equivalent over time. We hypothesize that these discrepancies may be attributed to the retrospective nature of the study conducted by SOUSA Correia J et al., which had a small sample size of 15 cases and a follow-up duration of only 3 months. Furthermore, the pain experienced with rectal cancer is of various types, with perineal neuralgia being just one aspect. It also includes invasive pain, inflammatory pain, and others. Real-world patients typically adhere to the WHO’s three-step therapy for cancer pain, which involves utilizing potent opioids like morphine and adjunctive medications such as pregabalin for analgesia [29].

Patients experiencing chronic pain frequently exhibit negative emotions, including depression and anxiety, which can intensify their perception of pain [30]. Previous research has identified the PHQ-9 as a significant evaluative tool for assessing perineal pain associated with depression, serving as a critical reference point for determining treatment efficacy [9]. In this study, a reduction in perineal pain was associated with a concomitant decrease in PHQ-9 scores, suggesting that pain serves as a precipitating factor for depression. Moreover, the effective alleviation of pain through radiofrequency therapy significantly contributes to the amelioration of psychological health issues secondary to pain [31].

Pain can lead to a decrease in sleep quality, and sleep disorders can also exacerbate pain. Chronic pain patients often have sleep disorders, such as decreased sleep efficiency and shortened sleep duration [32]. In this study, radiofrequency therapy significantly inhibited the PSQI, aligning with the observed trend in pain NRS scores. This clinical outcome parallels the findings of Zeyu Wu et al., who demonstrated that transdermal radiofrequency thermocoagulation effectively improved glossopharyngeal neuralgia and substantially reduced PSQI scores, thereby enhancing sleep quality [33].

## Conclusion

This study provides new insights regarding the clinical efficacy of two modes of CRF and H-PRF of the ganglion impar in the management of perineal pain in patients with advanced rectal cancer. Both treatment regimens could effectively control pain and improve the quality of life, with radiofrequency thermocoagulation showing superior long-term efficacy compared to high-voltage long-term pulsed radiofrequency.

LimitationsThis study is subject to several limitations. Firstly, it focuses solely on patients with rectal cancer experiencing perineal pain and utilizes various modes of ganglion impar radiofrequency. Given that perineal pain can have multiple etiologies, the efficacy of this treatment for pain stemming from other conditions remains uncertain. Secondly, the small sample size is a result of the limited availability of patients with rectal cancer and perineal pain. Additionally, the study did not conduct a robust calculation of the sample size based on epidemiological data of incidence rates with a larger sample size.

## Electronic supplementary material

Below is the link to the electronic supplementary material.

Supplementary Material 1

## Data Availability

The datasets used and/or analyzed during the current study are available from the corresponding author upon reasonable request.

## Abbreviations

GIB: Ganglion impar block
RF: Radiofrequency
CRF: Conventional radiofrequency
GSNP: Grading System for Neuropathic Pain
DSA: Digital subtraction angiography
BMI: Body Mass Index
NRS: Numerical Rating Scale
PHQ-9: Patient Health Questionnaire-9
PSQI: Pittsburgh Sleep Quality Index
IQR: Interquartile range
M: Median
